# The response of mesophyll conductance to short- and long-term environmental conditions in chickpea genotypes

**DOI:** 10.1093/aobpla/ply073

**Published:** 2018-12-11

**Authors:** Arjina Shrestha, Thomas N Buckley, Erin L Lockhart, Margaret M Barbour

**Affiliations:** 1 The Centre for Carbon, Water and Food, Faculty of Science, The University of Sydney, Sydney, Australia; 2 Department of Plant Sciences, University of California, Davis, CA, USA

**Keywords:** *Cicer arietinum*, mesophyll conductance, nitrogen-fixation, nitrogen nutrition, photosynthetic photon flux density

## Abstract

Abstract. Mesophyll conductance (*g*_m_) has been shown to vary between genotypes of a number of species and with growth environments, including nitrogen availability, but understanding of *g*_m_ variability in legumes is limited. We might expect *g*_m_ in legumes to respond differently to limited nitrogen availability, due to their ability to fix atmospheric N_2_. Using online stable carbon isotope discrimination method, we quantified genetic variability in *g*_m_ under ideal conditions, investigated *g*_m_ response to N source (N_2_-fixation or inorganic N) and determined the effects of N source and water availability on the rapid response of *g*_m_ to photosynthetic photon flux density (PPFD) and radiation wavelength in three genotypes of chickpea (*Cicer arietinum*). Genotypes varied 2-fold in *g*_m_ under non-limiting environments. N-fed plants had higher *g*_m_ than N_2_-fixing plants in one genotype, while *g*_m_ in the other two genotypes was unaffected. *g*_m_ response to PPFD was altered by N source in one of three genotypes, in which the *g*_m_ response to PPFD was statistically significant in N-fed plants but not in N_2_-fixing plants. There was no clear effect of moderate water stress on the *g*_m_ response to PPFD and radiation wavelength. Genotypes of a single legume species differ in the sensitivity of *g*_m_ to both long- and short-term environmental conditions, precluding utility in crop breeding programmes.

## Teaser

Given the growing interest in mesophyll conductance (*g*_m_) as a selection target for increased photosynthesis, and a lack of understanding of *g*_m_ regulation in legumes, we investigated the effect of water availability and nitrogen source on *g*_m_ in chickpea genotypes. We found that genotypes differed in their *g*_m_ sensitivity to nitrogen source, while there was no clear effect of reduced water availability on the *g*_m_ response to light intensity or quality. The significant variability in response of *g*_m_ to long- and short-term environmental conditions observed in our experiments indicates that inclusion of *g*_m_ as a selection trait is not straightforward.

## Introduction

Mesophyll conductance to CO_2_ (*g*_m_), which regulates the diffusion of CO_2_ from substomatal cavities to the sites of carboxylation, is now recognized as a significant and variable limitation to photosynthesis (Flexas *et al.* 2008, 2012). *g*_m_ is a combination of gaseous diffusion through the intercellular airspaces and diffusion in the liquid phase through the mesophyll cell walls, plasma membrane, cytosol and chloroplast envelope to chloroplast stroma (Evans *et al.* 2009). *g*_m_ has been shown to be influenced by different growth environments including water availability, photosynthetic photon flux density (PPFD), temperature, CO_2_ concentration and nitrogen nutrition (Warren *et al.* 2007; Flexas *et al.* 2008; Loreto *et al.* 2009; Bunce 2010; Douthe *et al.* 2011; Perez-Martin *et al.* 2014; Xiong *et al.* 2015; Olsovska *et al.* 2016). *g*_m_ variability within and among species and in response to growth conditions has been associated with leaf structure and anatomical properties, particularly the surface area of chloroplasts exposed to the intercellular spaces (*S*_c_), cell wall and chloroplast thickness (Evans *et al.* 2009; Tosens *et al.* 2012; Tomás *et al.* 2013), but see (Hanba *et al.* 2002; Tomás *et al.* 2014; Shrestha 2017). *g*_m_ variability may also result from the changes in leaf enzymatic processes including membrane permeability through aquaporins, AQPs (Terashima and Ono 2002; Hanba *et al.* 2004; Flexas *et al.* 2006, 2008, 2012) and CO_2_/bicarbonate equilibration though carbonic anhydrase, CA (Gillon and Yakir 2000; Perez-Martin *et al.* 2014; Momayyezi and Guy 2017). *g*_m_ has been suggested as an appropriate selection target to improve crop water-use efficiency (Flexas *et al.* 2013) while maintaining photosynthetic rate. An increase in *g*_m_ will increase chloroplastic CO_2_ concentration, and so increase photosynthetic rates, with no simultaneous increase in transpiration (assuming *g*_m_ and *g*_s_ can be decoupled; Barbour *et al.* 2010).

Grain legumes have received less attention than cereals in studies of *g*_m_ regulation. Unlike other plants, legumes can derive some of their nitrogen from symbiotic nitrogen-fixation in their root nodules (Graham and Vance 2003; Foyer *et al.* 2016). Nitrogen acquisition by these methods has been shown to differ in metabolic and transport processes (Schubert 1995), and studies have reported a higher energetic cost of symbiotic nitrogen-fixation compared to that of soil mineral N uptake and assimilation (Pate *et al.* 1979; Chapin *et al.* 1987; Andrews *et al.* 2009). Nitrogen source has also been shown to affect stomatal conductance (*g*_sw_; but not intercellular CO_2_ concentration) and photorespiratory rates, with lower *g*_sw_ and higher photorespiratory flux in NO_3_^−^-fed plants than in N_2_-fixing plants (Frechilla *et al.* 1999). Busch *et al.* (2018) recently showed that NO_3_^−^ assimilation via the photorespiratory pathway can increase the rate of CO_2_ assimilation by fixing carbon as amino acids, highlighting the intrinsic link between C and N metabolism in leaves. N_2_-fixing plants have also been reported to have higher leaf area per unit dry weight than NO_3_^−^-fed plants (Frechilla *et al.* 1999). Previous studies have reported a significant correlation between leaf anatomy (e.g. leaf thickness, leaf mass per area) and *g*_m_ (Syvertsen *et al.* 1995; Hanba *et al.* 1999). It is likely that different source of N nutrition could influence *g*_m_ through modifications in leaf anatomy or N assimilation processes. However, there are no reports to date whether nitrogen source influences *g*_m_.

Mesophyll conductance has also been found to respond to short-term changes in environmental conditions such as temperature and CO_2_ concentration (Flexas *et al.* 2008; von Caemmerer and Evans 2015; Xiong *et al.* 2015); however, there are conflicting results between studies regarding the short-term response of *g*_m_ to light environment. Positive relationships between *g*_m_ and PPFD have been observed in some studies (Gorton *et al.* 2003; Flexas *et al.* 2007; Douthe *et al.* 2011, 2012; Xiong *et al.* 2015, 2018) but not in others (Tazoe *et al.* 2009; Yamori *et al.* 2010). Théroux-Rancourt and Gilbert (2017) demonstrated that *g*_m_ response to PPFD is controlled by anatomical structure across the leaf profile highlighting the 3D nature of *g*_m_. Further, there has been speculation that rapid changes in *g*_m_ with PPFD are methodological artefacts (Tholen *et al.* 2012; Gu and Sun 2014). The two most commonly used methods for estimating *g*_m_ are (i) gas exchange in combination with ^13^C isotope discrimination (Evans *et al.* 1986), and (ii) gas exchange in combination with chlorophyll fluorescence (Harley *et al.* 1992). Both methods rely on models for the calculation of *g*_m_ and are sensitive to variation in the values of the model parameters (Pons *et al.* 2009). Studies examining the importance of growth environments (e.g. water and nitrogen limitation) on the sensitivity of *g*_m_ to light environment in different species and genotypes would be valuable to our understanding of *g*_m_ regulation. Xiong *et al.* (2015) found that the rapid responses of *g*_m_ to changes of CO_2_ concentration, temperature and PPFD were affected by nitrogen supplements in rice, and Barbour and Kaiser (2016) reported genotypic variation in the *g*_m_ response to nitrogen and water availability in wheat.

The present study was undertaken to investigate *g*_m_ regulation under a range of growth and environmental conditions in chickpea (*Cicer arietinum*). Chickpea is the second most important grain legume crop in terms of area and production globally (FAOSTAT 2014). Chickpea genotypes have been shown to differ in leaf gas exchange under ideal growth conditions (Mafakheri *et al.* 2010), but *g*_m_ variability has not yet been quantified in chickpea. In the present study, we attempted to address three questions: (i) Do chickpea genotypes differ in mesophyll conductance? (ii) Does the source of N influence *g*_m_ in chickpea and are there genotypic differences in this effect? (iii) Are there genotypic differences in the growth environment effects on the *g*_m_ response to PDF and radiation wavelength? Three experiments were conducted to answer these questions. The first experiment characterized *g*_m_ variability in 20 chickpea genotypes under controlled conditions. In the second experiment, three chickpea genotypes were grown employing either N_2_-fixation or inorganic nitrogen and measured under a range of PPFD. The third experiment examined the interactive effects of water availability and short-term changes in PPFD and radiation wavelength on *g*_m_ in three chickpea genotypes.

## Methods

### Plant material and experimental arrangements

#### Experiment 1: screening for g_m_ under non-limiting environments

Twenty genotypes of chickpea were grown in a controlled-environment growth room at the University of Sydney, Centre for Carbon Water and Food (Camden, NSW, Australia). Seeds were sown in 7 L pots filled with commercial potting mix supplemented with slow release fertilizer (Osmocote Exact, Scotts, NSW, Australia). Plants were maintained at 25 °C/17 °C in a 16-h photoperiod, 75 % relative humidity with irradiance (PPFD) of ~600 µmol m^−2^ s^−1^ at the top of the canopy. All plants were well-watered and fertilized throughout the experiment. Genotypes were sourced from: NSW Department of Primary Industries (DPI: Amethyst, Genesis 079, Kyabra, Jimbour and Yorker); NSW DPI in conjunction with Pulse Breeding Australia (PBA Hattrick, PBA Monarch and PBA Slasher); the WA Department of Agriculture and Fisheries (DAF: Sonali); the QLD DAF (Tyson) and ICARDA (Flip079C). In addition, nine breeding lines (BL1–9) were included which were sourced from the germplasm store at the University of Sydney Narrabri Campus. Of the 20 genotypes, 17 were desi and 3 kabuli **[see****Supporting Information—Table S1****]**. Desi types have small, dark, angular seeds, whereas kabuli types have large, rounded, light-coloured seeds (Leport *et al.* 2006).

#### Experiment 2: nitrogen source × PPFD × genotype

The nitrogen experiment was carried out on 3 of the 20 chickpea genotypes from the screening experiment; Flip079C and PBA Slasher and Sonali. The genotypes were selected based on their phenological similarity (all three genotypes are early varieties; C. Blessing, the University of Sydney, pers. comm.) so that physiological measurements could be made at the same growth stage. Flip079C belongs to kabuli type while PBA Slasher and Sonali are desi type. PBA Slasher and Sonali are parental genotypes in mapping population (A. L. Pattison, the University of Sydney, pers. comm.). The study was conducted in a controlled growth room with environmental condition similar to Experiment 1, except PPFD was 200 µmol m^−2^ s^−1^ at plant height. Plants were grown in 7 L pots, filled with washed river sand (N-free media) and lined with ~2.5 cm of gravel on the bottom of the pots. Five seeds were sown per pot and thinned to two seedlings per pot after 2 weeks. The two nitrogen source treatments were (i) inoculated with a peat-based Nodule N Rhizobium without mineral N supply (N_2_-fixing) and (ii) uninoculated and supplied with 2.5 mM NH_4_NO_3_ (N-fed).

The plants in both treatments were provided with quarter-strength modified Herridge N-free mineral nutrient solution (Herridge 1977): 250 µM CaCl_2_·2H_2_O, 250 µM KCl, 125 µM KH_2_PO_4_, 125 µM K_2_HPO_4_, 500 µM MgSO_4_·7H_2_O, 25 µM FeEDDHA and 25 µM Trace Elements (2.86 mg L^−1^ H_3_BO_3_, 1.81 mg L^−1^ MnCl_2_·4H_2_O, 0.11 mg L^−1^ ZnCl_2_; 0.05 mg L^−1^ CuCl_2_·2H_2_O; 0.025 mg L^−1^ Na_2_MoO_4_·2H_2_O). For the first 10 days after planting, 0.5 mM KNO_3_ was included in the Herridge nutrient solution for both treatments to help the plants establish. All the pots were then flushed with pure water to wash away any nitrogen residues from the media. Thereafter, inoculated plants received the N-free Herridge solution while the uninoculated plants received 2.5 mM NH_4_NO_3_ in addition to the Herridge solution. The pots in each N treatment (three genotypes × three pots × two replicate plants per pot) were placed on separate benches to avoid mixing of the throughfall waters and contamination of uninoculated pots. All the plants were watered with the nutrient solution in excess to avoid water stress at all times.

#### Experiment 3: water availability × PPFD × radiation wavelength × genotype

We used 3 of the 20 chickpea genotypes from the screening experiment: Amethyst, PBA Slasher and Sonali for the water availability experiment. PBA Slasher and Sonali were identified as among the drought tolerant genotypes, whereas Amethyst (desi type) was drought susceptible based on the grain yield ranking and drought indices (Kaloki 2017). The highest yielding genotype under well-watered conditions was PBA Slasher followed by Sonali, whereas under water limited conditions, Sonali was the highest yielding genotype. Amethyst has the lowest *g*_m_ value (from Experiment 1). Seeds were germinated in 7 L pots filled with commercial potting mix supplemented with slow release fertilizer (Osmocote Exact, Scotts, NSW, Australia). Plants were grown in a controlled-environment growth room at the University of Sydney, Centre for Carbon, Water and Food (Camden, NSW, Australia). The growth room was set to 25 °C/17 °C day/night temperature, 75 % relative humidity, 700 µmol m^−2^ s^−1^ PPFD at plant height and 14-h photoperiod. After emergence, the plants were thinned to two per pot and were well-watered until two watering treatments were imposed. The pots in each watering treatment (three genotypes × three pots × two plants per pot) were arranged in a completely randomized design. The watering treatment was imposed at 18 days after planting (DAP) when all the plants were at the vegetative stage: (i) one-half of the plants were kept well-watered by daily watering (WW); and (ii) the other half were exposed to water stress (WS) by withholding water until the first sign of temporary leaf wilting. Midday leaf water potential (Ψ_leaf_) of upper fully expanded leaves was measured to monitor water stress using a Scholander pressure chamber (115, Soil Moisture Equipment, Santa Barbara, CA, USA) and following the precautions recommended by Turner (1988). Midday Ψ_leaf_ measurements were performed on lateral branches for each genotype.

At the temporary wilting point (at which the apical leaves wilted at midday but recovered overnight, which occurred 7 days after the start of the water stress treatment), average midday leaf water potentials for WW and WS plants were −0.6 and −1.2 MPa, respectively. The weight of each WS pot at this point was designated as the target weight for the pot. The soil moisture content of the WS pots was maintained gravimetrically throughout the measurement period (7 days) by weighing each pot daily at 1 h after the start of the light period and adding water to replace that transpired and evaporated.

### Simultaneous gas exchange and mesophyll conductance measurements

#### Experiment 1: screening for g_m_ under non-limiting environments

Gas exchange measurements and regulation of leaf environmental conditions were conducted using a Li-6400XT portable photosynthesis system (LI-COR Biosciences, Lincoln, NE, USA). Five weeks after sowing, each of five leaves per genotype were enclosed in 12 cm^2^ (2 × 6) clear-top chamber of the Li-6400XT fitted with a red-green-blue LED light source (Li-6400 18A) set to 1300 μmol m^−2^ s^−1^ (10 % blue and 90 % red). The uppermost fully expanded leaves of the primary branches were used for the measurements. Leaf area within the chamber was calculated from the digitized images of the leaf using ImageJ (NIH, Bethesda, MD, USA) and the gas exchange variables were recalculated with the corrected leaf area. CO_2_ concentration inside the chamber was fixed at 400 µmol mol^−1^, leaf temperature was set at 25 °C, and relative humidity was maintained between 70 and 80 %. CO_2_ concentration differences between the air entering and leaving the chamber were in the range of 31–105 to obtain the precise and accurate estimation of *g*_m_, considering the precautions recommended by Pons *et al.* (2009) for online isotope method. Data points with CO_2_ differentials <30 were excluded because of the associated error in the discrimination measurements. Kyabra genotype had one unrealistically high *g*_m_ value (>3 mol m^−2^ s^−1^ bar^−1^), and thus this data point was removed from ANOVA analysis. All the measurements were made at 21 % O_2_. Each leaf remained in the chamber for at least 30 min to allow time for the leaf to adjust to the chamber conditions before gas exchange and online discrimination measurements were made. Gas exchange was recorded at 1-min intervals.

Mesophyll conductance was estimated using the online carbon isotope discrimination method (Evans *et al.* 1986; Tazoe *et al.* 2009) for all the experiments. The Li-6400XT was coupled to a Tunable-Diode Laser Absorption Spectrometer (TDL, model TGA100A, Campbell Scientific, Inc., Logan, UT, USA), which measured the stable carbon and oxygen isotope compositions of CO_2_ (^13^CO_2_, C^18^O^16^O), as described by Barbour *et al.* (2007). Leaf chamber inlet and outlet air streams were subsampled to the TDL. Mesophyll conductance was estimated from the difference between calculated carbon isotope discrimination assuming infinite *g*_m_ (Δ^13^C_i_), and that measured by the coupled system (Δ^13^C_obs_), as described in Jahan *et al.* (2014), including the ternary corrections as described by Farquhar and Cernusak (2012).

$$\begin{array}{l} \begin{array}{ll} & \Delta^{13}C_{i}=\;\frac{1}{1-t}\;\left[a_{b\;}\frac{C_{a}-C_{s}}{C_{a}}+a_{s\;}\frac{C_{s}-C_{i}}{C_{a}}\right]\\ & \;\;\;+\frac{1+t}{1-t}\;\left[b\;\frac{C_{i}}{C_{a}}-\;\frac{{Α}_{b}}{{Α}_{\overset{´}{e}}}\overset{´}{e}\frac{R_{d}}{A+R_{d}}\;\frac{C_{i}-\Gamma^{*}}{C_{a}}-\frac{{Α}_{b}}{{Α}_{f}}f\frac{\Gamma^{*}}{C_{a}}\right] \end{array} \end{array}$$ (1)

where C_a_, C_s_ and C_i_ are the ambient, leaf surface and intercellular CO_2_ partial pressures, *a*_b_ and *a*_s_ are the fractionations during diffusion through the leaf boundary layer and the stomata, respectively, *b* is the fractionation associated with carboxylation, *f* is the fractionation associated with photorespiration, α_b_ is the fractionation factor for carboxylation (1 + *b*), α_é_ is the fractionation factor for day respiration (1 + *é*), α_f_ is the fractionation factor for photorespiration (1 + *f*). The assumed values for various fractionation factors during CO_2_ diffusion within the leaf, used for calculating *g*_m_ are shown in Table 1. *R*_d_ is the rate of day respiration and Γ* is the compensation point in the absence of *R*_d_. Both *R*_d_ and Γ* were predicted from leaf temperature using the approach described by Bernacchi *et al.* (2001). *R*_d_ is known to vary between genotypes of crop species (e.g. Jahan *et al.* 2014 found *R*_d_ varied between wheat cultivars), so in the absence of *R*_d_ measurements for the chickpea, we conducted a sensitivity analysis to determine the effect of errors in the *R*_d_ assumption. We assumed *R*_d_ was 1.5 μmol m^−1^ s^−1^ at 25 °C for all genotypes in all experiments. When *R*_d_ was varied between 1 and 2 mol m^−2^ s^−1^, *g*_m_ changed by 0.01–0.02 mol m^−2^ s^−1^ (2 %) for measurements made with red or red-blue light and by 0.02–0.03 mol m^−2^ s^−1^ with blue light (3 %). These negligible errors were deemed unlikely to alter conclusions drawn from the measurements.

**Table 1.** Fractionation factors used in the calculation of *g*_m_. *Fractionation associated with day respiration (*é*) was corrected for disequilibrium between growth CO_2_ δ^13^C (−14 ‰; measured by a stable isotope cavity ring down laser, G11101-i, Picarro, Santa Clara, CA, USA) and measurement CO_2_ δ^13^C (−31 ‰ for Experiment 1 and −4 ‰ for Experiments 2 and 3; measured by Tunable-Diode Laser Absorption Spectrometer; TDL, model TGA100A, Campbell Scientific, Inc., Logan, UT, USA).

	Symbol	Value (‰)	Reference
Fractionation during leaf boundary layer diffusion	*a*_b_	2.9	Evans *et al.* (1986)
Fractionation during stomata diffusion	*a*_s_	4.4	Farquhar and Richards (1984)
Fractionation during CO_2_ diffusion and dissolution	*a*_m_	1.8	O’Leary (1984)
Fractionation during carboxylation	*b*	30	Guy *et al.* (1993)
Fractionation during day respiration*	*e*	−3	Tcherkez *et al.* (2010)
Fractionation during photorespiration	*f*	16.2	Evans and von Caemmerer (2013)

In Equation (1), *t* is the ternary correction factor (Farquhar and Cernusak 2012), and is given by:

$$\begin{array}{l} t=\frac{\alpha_{ac}E}{2g_{ac}} \end{array}$$ (2)

where *E* is the transpiration rate (mmol m^−2^ s^−1^), α_ac_ is the fractionation factor of CO_2_ diffusion in air (1 + *ā*), *ā* is the weighted fractionation through the leaf boundary layer and stomata (Evans *et al.* 1986). *g*_ac_ denotes the total conductance to CO_2_ diffusion including the boundary layer and stomatal conductance.

Then, mesophyll resistance (*r*_m_ = 1/*g*_m_) is given by Farquhar and Cernusak (2012):

$$\begin{array}{l} r_{m}=\;\frac{1-t}{1+t}\;\left(\Delta^{13}C_{i}-\Delta^{13}C_{obs}\right)\frac{C_{a}}{A\left(b-a_{m}-\frac{\alpha_{b}}{\alpha_{\overset{´}{e}}}\overset{´}{e}\frac{R_{d}}{A+R_{d}}\right)} \end{array}$$ (3)

*A* is the CO_2_ assimilation rate (µmol m^−2^ s^−1^), *a*_m_ is the fractionation factor for liquid phase CO_2_ diffusion and dissolution (‰).

∆^13^C_obs_ is calculated from the following equation (Evans *et al.* 1986):

$$\begin{array}{l} \Delta^{13}C_{obs}=\frac{\xi (\Delta_{o}-\Delta_{e})}{1+\Delta_{o}-\xi (\Delta_{o}-\Delta_{e})} \end{array}$$ (4)

where

$$\begin{array}{l} \xi =\frac{C_{e}}{C_{e}-C_{o}} \end{array}$$ (5)

*C*_e_ and δ_e_ are concentrations and isotope compositions of CO_2_ of dry air entering the leaf chamber and *C*_o_ and δ_o_ are concentrations and isotope compositions of CO_2_ of dry air exiting the chamber, respectively. Carbon and oxygen isotope compositions of CO_2_ were obtained from the TDL.

Two calibration cylinders were used to calibrate the TDL, spanning the range in concentrations of the isotopologues of the leaf chamber inlet and outlet air streams. Total CO_2_ concentrations and isotope compositions of the calibration cylinders were measured using a stable isotope mass spectrometer at the National Institute of Water and Atmospheric Research, Wellington, New Zealand. Carbon isotope ratios are presented relative to the Vienna Pee Dee belemnite standard, and oxygen isotope ratios of CO_2_ and water vapour are presented relative to the Vienna Standard Mean Oceanic Water (VSMOW) standard. The TDL received standards from the cylinders every 6 min and the raw values of the sample air streams within this time period were calibrated against these standards. Interchanging between calibration cylinders and the sample air streams was enabled by a manifold regulated by a datalogger (CR3000, Campbell Scientific, Inc.).

#### Experiment 2: nitrogen source × PPFD × genotype

Leaf gas exchange and mesophyll conductance measurements were conducted 5 weeks after planting. The Li-6400XT was fitted with a custom-built leaf chamber of area 38 cm^2^ (Loucos *et al.* 2017) and red-green-blue light source (Li-6400 18A) for this experiment. The boundary layer conductance for the chamber was estimated using the method described in Barbour *et al.* (2007). To examine leaf responses to rapidly changing PPFD, simultaneous leaf gas exchange and isotopic discrimination measurements were made in the order 1000, 800, 600, 400, 300 μmol m^−2^ s^−1^, with the light colour was set to 10 % blue and 90 % red. The measurements were made for plants in both N treatments and leaves remained in the chamber for at least 15 min at each irradiance. Throughout the measurements, CO_2_ concentration in the sample cell was maintained at 400 µmol mol^−1^, flow rate at 500 μmol s^−1^ and leaf temperature at 25 °C. CO_2_ concentration differences between the air entering and leaving the chamber were in the range of 40–90 (corresponding to the lowest and the highest PPFD, respectively). All the measurements were made at 21 % O_2_.

#### Experiment 3: water availability × PPFD × radiation wavelength × genotype

Leaf gas exchange and mesophyll conductance measurements were performed as for Experiment 2, except that PPFD was set at (in order) 950, 700 and 400 μmol m^−2^ s^−1^, under red radiation and then under blue radiation. The blue radiation had a peak emission at 457 nm, with a range from 424 to 524 nm, while the red radiation peak emission was centred at 636 nm, ranging from 584 to 661 nm. The leaves remained in the chamber for at least 15 min at each ‘PPFD-wavelength’ step. The measurements were made for both the well-watered and water-stressed plants at 21 % O_2_. CO_2_ concentration differences between the air entering and leaving the chamber were in the range of 37–148 (for the lowest intensity of blue radiation to the highest intensity of red radiation, respectively). Leaf water potential (Ψ_leaf_) was measured for all leaves immediately after gas exchange measurements.

### Crop traits

In the nitrogen source experiment (Experiment 2), the youngest fully expanded leaf samples were collected after the gas exchange measurements and were oven-dried at 65 °C for 72 h. Samples were then ground to a fine powder and analysed for total N content (N%) and ^15^N composition using isotope ratio mass spectrometry (Delta V, Thermo Fisher Scientific, Bremen, Germany). The plants were harvested, cleaned of sand and roots were washed. Roots and nodules were separated and oven-dried at 65 °C for 72 h for measurement of dry weight. The proportion of N derived from N-fixation (%Ndfa) for the N-fed plants was determined using the δ^15^N Natural Abundance Method (Unkovich *et al.* 2008).

$$\begin{array}{l} \%Ndfa=\;\frac{\delta^{15}N\;of\;soil\;N-\delta^{15}N\;of\;N_{2}\text{-}fixing\;legume}{\delta^{15}N\;of\;soil\;N-\delta^{15}N\;of\;N_{2}\;}\;\times \frac{100}{1} \end{array}$$ (6)

where δ^15^N of N_2_-fixing legume represents the δ^15^N value of the non-inoculated legume supplied with NH_4_NO_3_, and δ^15^N of N_2_ is the δ^15^N value of the inoculated legume grown with atmospheric N_2_ as the sole source of N. δ^15^N of soil N (NH_4_NO_3_ fertilizer supplied to N-fed plants) was estimated using isotope ratio mass spectrometry.

### Statistical analyses

Significant differences between values were assessed using general analysis of variance, as implemented by GenStat 14th edition (VSN International Ltd, London, UK), and means were compared using Fisher’s unprotected least significant difference test. Differences were considered statistically significant when *P* < 0.05.

## Results

### Do chickpea genotypes differ in mesophyll conductance?

**Figure 1.**
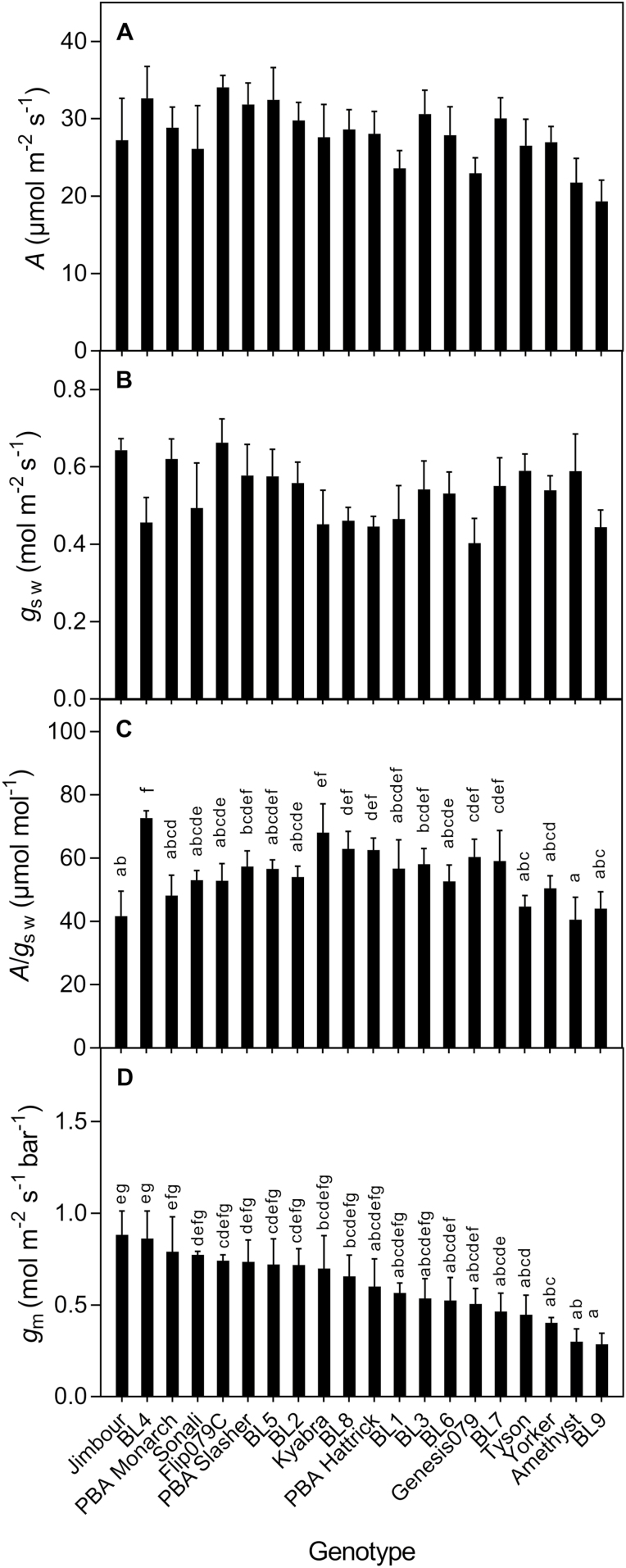
Photosynthetic rate (*A*; A), stomatal conductance to water vapour (*g*_sw_; B), leaf-intrinsic water use efficiency (*A/g*_sw_; C) and mesophyll conductance (*g*_m_; D) of 20 chickpea genotypes grown and measured under non-limiting controlled environmental conditions. Mean and SE are shown (*n* = 3–5). Letters indicate significant differences (*P* < 0.05) between genotypes.

The screening experiment results showed ~1.7-fold range in net photosynthetic rate (*A*) and stomatal conductance to water vapour (*g*_sw_) among the 20 chickpea genotypes, while *g*_m_ ranged >2-fold from 0.29 to 0.88 mol m^−2^ s^−1^ bar^−1^ (BL9 and Jimbour, respectively; Fig. 1 and see **Supporting Information–TableS3**). Average leaf intrinsic water-use efficiency (*A*/*g*_sw_) varied between 40 (BL9) and 73 μmol mol^−1^ (BL4), and was positively, but weakly, related to *g*_m_ (*A*/*g*_sw_ = 22.1 + 41.1*g*_m_, *R*^2^ = 0.25, *P* = 0.023, data not shown). Genotypic differences in *A* and *g*_sw_ were not statistically significant, but *g*_m_ and *A*/*g*_sw_ differed significantly between genotypes (*P* = 0.023 and *P* = 0.011, respectively; Fig. 1). In water availability and nitrogen source experiments, Sonali had significantly higher average *g*_m_ than the other genotypes (Amethyst, PBA Slasher and Flip079C) when grown and measured under ideal conditions.

### Does the source of N influence *g*_m_ in chickpea and are there genotypic differences in this effect?

Three of the 20 chickpea genotypes (Flip079C, PBA Slasher and Sonali) were used to compare *g*_m_ of uninoculated, N-fed (2.5 mM NH_4_NO_3_) plants with that of inoculated, N_2_-fixing plants. Some nodulation was observed in uninoculated, N-fed plants (Fig. 5). However, the nodule size and nodule number in N-fed plants was less than one-twentieth than that in N_2_-fixing plants (*P* < 0.001, df = 14). Leaves of N_2_-fixing plants were depleted in ^15^N compared to N-fed leaves (*P* < 0.001; genotype averages: 1.8 ± 0.2 ‰ N-fed and −1.8 ± 0.09 ‰ for N_2_-fixing leaves) indicating that different nitrogen sources were used. The δ^15^N value of NH_4_NO_3_ fertilizer supplied to N-fed plants was 2.4 ‰. N-fed PBA Slasher and N-fed Sonali had δ^15^N values close to that of the fertilizer indicating negligible N derived from N-fixation (%Ndfa). %Ndfa for PBA Slasher and Sonali was 6.2 and 9.3 %, respectively. The δ^15^N value of N-fed Flip079C (1.3 ‰) was lower (*P* = 0.01) than that of the N fertilizer and so the proportion of N derived from N-fixation was higher, at 25 %.

**Figure 2.**
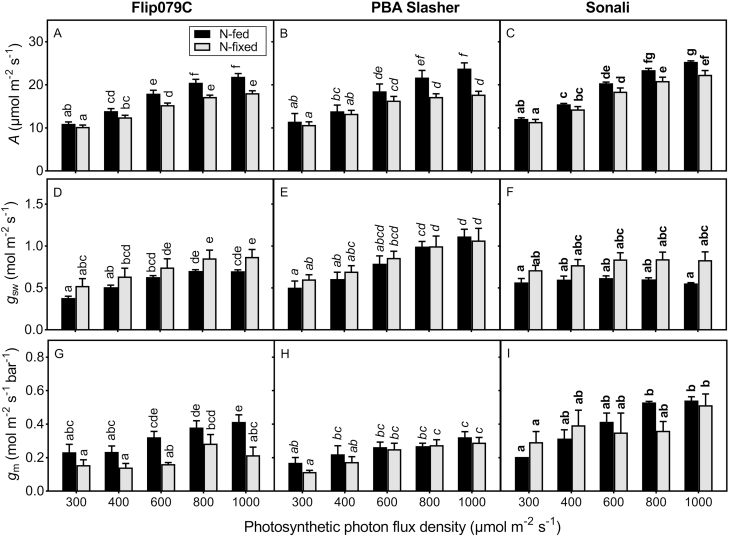
Photosynthetic rate (*A*; A, B, C), stomatal conductance to water vapour (*g*_sw_; D, E, F) and mesophyll conductance (*g*_m_; G, H, I) of three chickpea genotypes grown under two nitrogen source treatments and measured under different photon flux densities. Means and SE are shown (*n* = 5–6). Letters indicate significant differences (*P* < 0.05) between the treatments within each genotypes.

N-fed plants had higher photosynthetic rates than N_2_-fixing plants when measured at high PPFD across the three genotypes (Fig. 2). *g*_sw_ was higher for N_2_-fixing plants than for N-fed plants but the differences were not significant at each PPFD (Fig. 2). Interestingly, there was a significant interactive effect of genotype by nitrogen source (*P* = 0.017) for *g*_m_ (Table 2; Fig. 2 and **seeSupporting Information–TableS4**). N_2_-fixing Flip079C plants had lower *g*_m_ values than N-fed Flip079C plants and the difference was significant at higher PPFD. However, nitrogen source did not affect *g*_m_ in PBA Slasher and Sonali. The chloroplastic CO_2_ concentration (*C*_c_) was not affected by nitrogen source for any genotype.

**Table 2.** Effects of PPFD, nitrogen source and genotypes on net photosynthetic rate (*A*), stomatal conductance to water vapour (*g*_sw_) and mesophyll conductance to CO_2_ (*g*_m_). The degree of freedom (df) for PPFD = 4, nitrogen source = 1 and genotypes = 2.

		*A*	*g*_sw_	*g*_m_
PPFD	*F*	160.16	15.71	16.06
	*P*	<0.001	<0.001	<0.001
Nitrogen source	*F*	61.28	19.99	14.67
	*P*	<0.001	<0.001	<0.001
Genotypes	*F*	23.04	8.88	32.86
	*P*	<0.001	<0.001	<0.001
PPFD × nitrogen source	*F*	4.94	NS	NS
	*P*	0.001	NS	NS
PPFD × genotypes	*F*	NS	2.55	NS
	*P*	NS	0.014	NS
Nitrogen source × genotypes	*F*	NS	2.77	4.26
	*P*	NS	0.067	0.017
PPFD × nitrogen source × genotypes	*F*	NS	NS	NS
	*P*	NS	NS	NS

**Figure 3.**
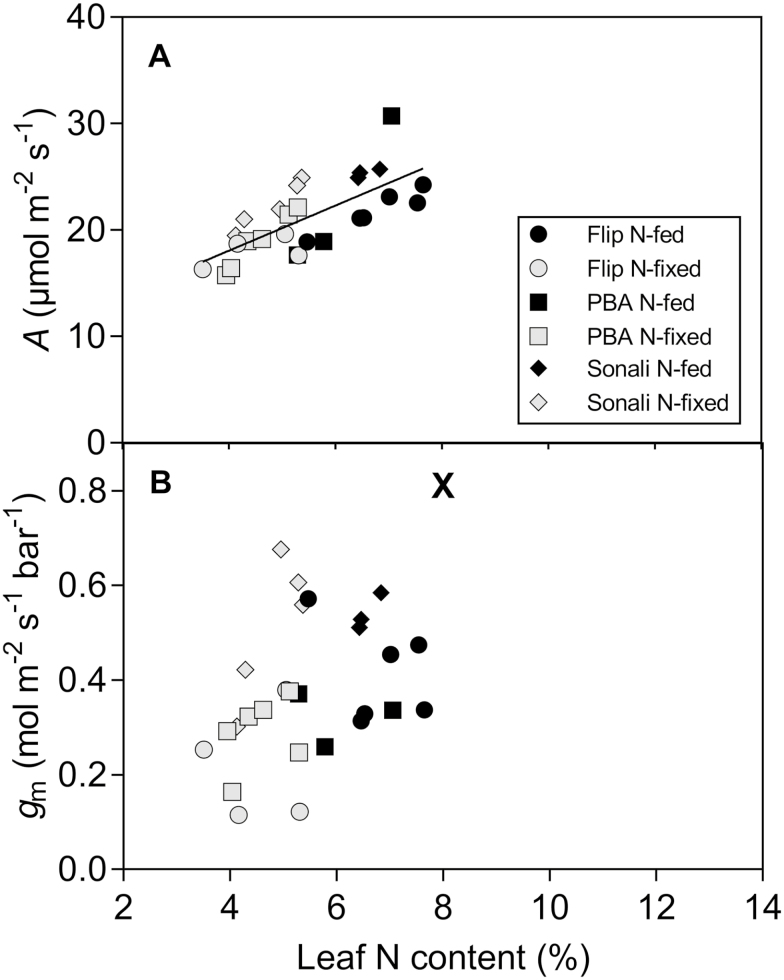
Relationships between leaf N content and photosynthetic rate (*A*; A) and mesophyll conductance to CO_2_ (*g*_m_; B), measured at 1000 µmol m^−2^ s^−1^ PPFD, for three chickpea genotypes grown under two nitrogen source treatments. The solid line in plot A indicates a significant linear regression (*P* < 0.001, *R*^2^ = 0.51).

Leaf N content (%N) was affected by the nitrogen source (*P* < 0.001) and was significantly lower for N_2_-fixing (4.6 %) than for N-fed plants (6.5 %). The relationships between %N and *A* were positive when all the data were pooled together (*P* < 0.0001, *R*^2^ = 0.51) (Fig. 3). However, we did not find any relationship between *g*_m_ and %N (Fig. 3).

### Are there genotypic differences in the growth environment effects on the *g*_m_ response to PPFD and wavelength?

*g*_m_ response to PPFD was assessed in N-fed and N_2_-fixing plants of three genotypes (Flip079C, PBA Slasher and Sonali). Table 2 shows the result of the ANOVA. Our results showed genotypic differences in the effect of N source on the *g*_m_ sensitivity to PPFD **[see****Supporting Information—Table S2****]**. The linear relationships between *g*_m_ and PPFD (regression fitted to the individual data) were significant for N-fed plants of each genotype (Flip079C: *P* < 0.001; PBA Slasher: *P* = 0.004; Sonali: *P* < 0.001), while in N_2_-fixing plants, the linear relationship between *g*_m_ and PPFD was significant for PBA Slasher (*P* < 0.001) and Flip079C (*P* = 0.038) but not for Sonali (*P* > 0.05).

**Figure 4.**
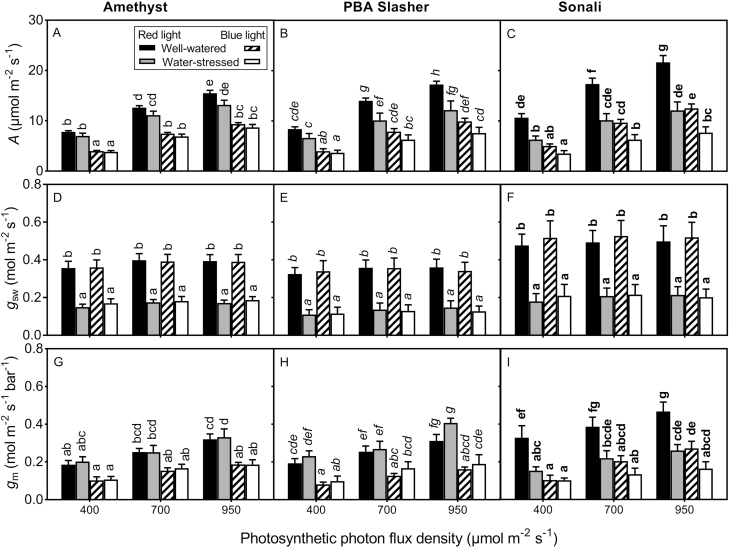
Photosynthetic rate (*A*; A, B, C), stomatal conductance to water vapour (*g*_sw_; D, E, F) and mesophyll conductance (*g*_m_; G, H, I) of three chickpea genotypes grown under well-watered or water-stressed conditions and measured under varying photon flux density and radiation wavelength. Means and SE are shown (*n* = 5–6). Letters indicate significant differences (*P* < 0.05) between the treatments within each genotypes.

Three of the 20 genotypes (Amethyst, PBA Slasher and Sonali) were examined for the effect of water availability on the short-term response of *g*_m_ to PPFD and wavelength (Table 3 and **see Supporting Information–TableS5**). Water stress lowered leaf water potential, Ψ_leaf_ (*P* < 0.001). The average midday Ψ_leaf_ for WW and WS plants were −0.66 and −1.32 MPa, respectively, i.e. the WS plants were moderately stressed, but we did not find genotypic differences in Ψ_leaf_. *g*_m_ decreased linearly with decreasing PPFD but the *g*_m_ reduction was not significant for the water-stressed PBA Slasher, water-stressed Sonali measured under blue radiation and well-watered Sonali under red radiation (*P* > 0.05; Fig. 4) **[see****Supporting Information—Table S2****]**.

**Table 3.** Effects of PPFD, radiation wavelength, water stress and genotypes on net photosynthetic rate (*A*), stomatal conductance to water vapour (*g*_sw_) and mesophyll conductance to CO_2_ (*g*_m_). The degree of freedom (df) for PPFD = 2, wavelength = 1, water stress = 1 and genotypes = 2.

		*A*	*g*_sw_	*g*_m_
PPFD	*F*	205.78	NS	41.43
	*P*	<0.001	NS	<0.001
Wavelength	*F*	365.35	NS	157.79
	*P*	<0.001	NS	<0.001
Water stress	*F*	120.97	250.92	5.96
	*P*	<0.001	<0.001	0.016
Genotypes	*F*	10.7	20.32	3.18
	*P*	<0.001	<0.001	0.044
PPFD × wavelength	*F*	6.19	NS	NS
	*P*	0.003	NS	NS
PPFD × water stress	*F*	8.64	NS	NS
	*P*	<0.001	NS	NS
Wavelength × water stress	*F*	20.02	NS	2.61
	*P*	<0.001	NS	0.10
PPFD × genotypes	*F*	NS	NS	NS
	*P*	NS	NS	NS
Wavelength × genotypes	*F*	NS	NS	NS
	*P*	NS	NS	NS
Water stress × genotypes	*F*	21.57	3.62	22.72
	*P*	<0.001	0.029	<0.001
Wavelength × water stress × genotypes	*F*	2.31	NS	4.92
	*P*	0.1	NS	0.008

Switching from red radiation to blue radiation while maintaining constant PPFD reduced *A* and *g*_m_ but not *g*_sw_ in both WW and WS plants of the three genotypes (Table 3; Fig. 4). There was also a significant interactive effect of genotype by water stress by radiation wavelength for *g*_m_ (*P* = 0.008; Table 3; Fig. 4). Water stress reduced *g*_m_ only in Sonali when measured under red radiation. *g*_m_ was unaffected by water availability under blue radiation in Sonali and under any radiation wavelength in Amethyst and PBA Slasher.

## Discussion

### Mesophyll conductance varies between genotypes

*g*_m_ has been recognized as a significant and variable limitation to photosynthesis in a range of species, but there is limited information on *g*_m_ variability in legumes including chickpea. The 20 genotypes screened here showed a significant difference in *g*_m_ values. Genotypic variation in *g*_m_ has been reported for cereals (Centritto *et al.* 2009; Barbour *et al.* 2010; Gu *et al.* 2012; Jahan *et al.* 2014), a few other crop species (Lauteri *et al.* 1997; Galmés *et al.* 2011; Tomás *et al.* 2014) and recently among soybean edamame genotypes (Tomeo and Rosenthal 2017), faba and field pea genotypes (Shrestha 2017). We did not observe any clear differences in *g*_m_ values between the two types of chickpea (desi or kabuli) under non-limiting growth conditions. Barbour *et al.* (2016) reported the first hints of genetic control of *g*_m_ in bread wheat. Genotypic variation in *g*_m_ values in chickpea in our study might be due to the leaf anatomical or biochemical differences (not evaluated in the current study) between the genotypes.

### When N-fixation is the sole source of plant N, *g*_m_ is reduced in one genotype but not in two others

The current study showed that chickpea genotypes differed in their *g*_m_ response to nitrogen source. The genotype Flip079C had higher *g*_m_ when fertilized with nitrogen than when nitrogen was fixed by *Rhizobium inocula*; however, nitrogen source did not affect *g*_m_ in PBA Slasher and Sonali. Conversely, genotypes responded similarly to nitrogen source in terms of photosynthetic rate and leaf N content. Leaf N content was significantly lower for N_2_-fixing than for N-fed plants, as reported by Lodeiro *et al.* (2000) in common beans. We found a significant positive correlation between *A* and leaf N content, as reported in many other studies (Evans 1989; Reich *et al.* 1994; Li *et al.* 2009; Yamori *et al.* 2010), due to the dependence of photosynthesis on nitrogenous compounds (but see Adams *et al.* 2016). A higher photorespiratory flux in NO_3_^−^-fed plants than in N_2_-fixing plants was reported by Frechilla *et al.* (1999) and Busch *et al.* (2018) showed that NO_3_^−^ assimilation via the photorespiratory pathway can increase the rate of CO_2_ assimilation. However, the results of our study suggest that inorganic N source allowed higher assimilation through higher leaf N content.

There are no published studies on variability of *g*_m_ between N_2_-fixing and inorganic N-fed legumes; nevertheless, reduced nitrogen availability has been shown to reduce *g*_m_ in several species (Warren 2004; Bown *et al.* 2009; Li *et al.* 2012; Xiong *et al.* 2015). The mechanism of *g*_m_ regulation under different nitrogen sources is unclear. *g*_m_ response to nitrogen availability has been shown to be strongly correlated to *S*_c_ (Xiong *et al.* 2015) and chloroplast size (Li *et al.* 2012). Leaf ultrastructural properties of the genotypes were not examined in this study, and future work should investigate genotypic variation in leaf anatomy to understand the regulation of *g*_m_ in response to these growth conditions. Regarding the biochemical component of *g*_m_, Warren (2004) suggested that a correlation between nutrient supply and abundance or activity of CA and/or AQPs seems unlikely since CA and AQPs have a very low N cost. On the other hand, several studies have shown that AQP gene expression in the root system (Clarkson *et al.* 2000; Guo *et al.* 2007; Ishikawa-Sakurai *et al.* 2014; Ren *et al.* 2015) or in the stem xylem (Hacke *et al.* 2010) is affected by nitrogen supply and/or nitrogen forms in the medium. Whether *g*_m_ is limited by nitrogen investment in one or more enzymes or membrane proteins remains to be investigated. In the current study, we did not find any relationship between leaf N content and *g*_m_, consistent with previous studies reporting weak N–*g*_m_ relationships (Warren 2004; Barbour and Kaiser 2016). Higher *g*_m_ in N-fed Flip079C could simply reflect the relationship between *A* and *g*_m_ (*P* < 0.001, *R*^2^ = 0.64, data not shown). Further, the chloroplastic CO_2_ concentration (*C*_c_) was not affected by nitrogen source, suggesting that mesophyll limitation may not be responsible for the lower photosynthetic rate in N_2_-fixing plants.

**Figure 5.**
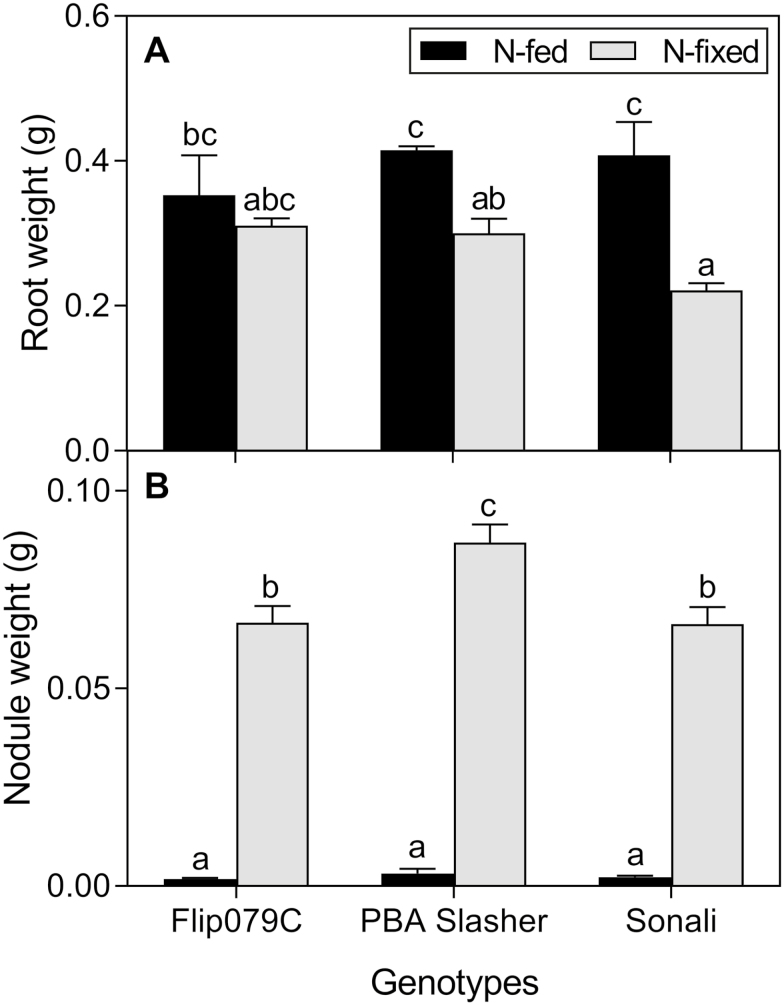
Root (A) and nodule weight (B) of three chickpea genotypes grown under two nitrogen source treatments. Means and SE are shown (*n* = 5–6). Letters indicate significant differences (*P* < 0.05) between the treatments.

It is not clear how nitrogen source could affect *g*_m_ in some genotypes but not in others. Flip079C is a kabuli chickpea and PBA Slasher and Sonali belong to the desi group. Studies have shown that the two types differ in morphology, nutrition and response to abiotic stresses (Porta-Puglia *et al.* 2000; Walley *et al.* 2005; Leport *et al.* 2006; Purushothaman *et al.* 2014; Imran *et al.* 2015). The gene pools for desi and kabuli types have been separate for many years (Gowda *et al.* 1987; Porta-Puglia *et al.* 2000) and genes associated with *g*_m_ may differ between the two types. It would be interesting to elucidate whether the genotypic difference observed here is related to the types of chickpea. The proportion of N derived from N-fixation (%Ndfa) was higher for N-fed Flip079C than for N-fed PBA Slasher and Sonali. N_2_-fixing plants had reduced root biomass compared to N-fed plants in PBA Slasher and Sonali, but nitrogen source had no effect on the root biomass of Flip079C (Fig. 5). von Caemmerer and Evans (2015) observed that the temperature response of *g*_m_ differed greatly between species, and proposed that variation in the *g*_m_ response may be due to variation in the activation energy for membrane permeability to CO_2_ (AQPs) and the effective path length for liquid phase diffusion (cell wall thickness). Future studies should investigate genotypic differences in leaf anatomy, enzymatic processes and the role of photorespiration in carbon and nitrogen assimilation under different sources of N nutrition (Busch *et al.* 2018).

Despite a lack of clear understanding of the underlying mechanisms of *g*_m_ regulation under different nitrogen sources, the observed genotypic variation in *g*_m_ sensitivity is interesting in the context of the recognized importance of legume-based farming systems and thus warrants further research.

### The *g*_m_ response to PPFD and radiation wavelength varies between genotypes and with water and N availability

In the present study, *g*_m_ significantly differed only between the highest and the lowest PPFD with an average change of ≈40 % between 950 and 400 µmol m^−2^ s^−1^ in the water availability experiment (Experiment 3), and an average change of ≈48 % between 1000 and 300 µmol m^−2^ s^−1^ in the nitrogen source experiment (Experiment 2). The sensitivity of the PPFD response in our study was different from that observed by Douthe *et al.* (2011, 2012) in *Eucalyptus* species. They found a positive relationship between *g*_m_ and PPFD at low intensities (i.e. when PPFD was lowered from 600 or 500 to 200 µmol m^−2^ s^−1^) but no change in *g*_m_ at higher intensities. The dissimilarity in results may be related to species-specific differences or to differences in growth environments.

*g*_m_ response to PPFD was altered by nitrogen source in only one of three genotypes, Sonali, in which the *g*_m_ response to PPFD was statistically significant in N-fed plants but not in N_2_-fixing plants. However, the response of *A* to PPFD was significant for both N-fed and N_2_-fixing plants in all three genotypes. Xiong *et al.* (2015) reported that the *g*_m_ response to PPFD differed with N supplement in rice, with *g*_m_ increasing with PPFD in high N leaves while remaining unaffected in low N leaves, suggesting an important role of N in rapid response of *g*_m_. We are unable to explain, on the basis of the present results, the cause of the observed genotypic variability in the N source effect on *g*_m_–PPFD relationships. The mechanism of *g*_m_ response to short-term changes in PPFD is not yet clear. Rapid responses of *g*_m_ to environmental factors have been attributed to CA and AQPs. Transcript abundance of two AQP isoforms has been shown to substantially up-regulated by radiation within minutes in *Juglans regia* (Cochard *et al.* 2007; Baaziz *et al.* 2012). Day respiration has been shown to be influenced by the source of nitrogen (NH_4_^+^ or NO_3_^−^) supplied to plants (Guo *et al.* 2005). The link between PPFD and day respiration (Noguchi 2005) and nitrogen source might have played some role in the N source effect on the apparent *g*_m_–PPFD relationship, through the influence of respiratory fractionation on *g*_m_ estimates (Barbour *et al.* 2017).

The present study showed no general trend in the effects of water availability on the *g*_m_–PPFD relationships. However, the response of *g*_m_ to PPFD was not significant for the water-stressed PBA Slasher and water-stressed Sonali when they were measured under blue radiation. All genotypes responded similarly to radiation wavelength under both WW and WS conditions. The reduction in *A* and *g*_m_ when leaves were exposed to blue radiation compared to red radiation of the same intensity was similar to reductions reported in previous studies in *Nicotiana tabacum*, *Platanus orientalis* (Loreto *et al.* 2009), *Populus* × *canadensis* and *Quercus ilex* (Pallozzi *et al.* 2013). *g*_m_ was measured using chlorophyll fluorescence-based methods in these two studies and Loreto *et al.* (2009) demonstrated that the *g*_m_ response to blue light is real, although approximately half of the observed effect of blue radiation on *g*_m_ might be attributable to experimental artefacts. Nevertheless, the fact that two methods that rely on substantially different assumptions produce similar results supports the hypothesis that the response of *g*_m_ to radiation wavelength is real. Further, differential response of *g*_m_ and *g*_sw_ to radiation wavelength in our study suggest uncoupling of the two conductance in the studied genotypes and environmental conditions, as also observed under blue radiation by Loreto *et al.* (2009) and under water stress conditions by Bunce (2009) but in contrast to the usually coregulation observed in wider multispecies data sets (Flexas *et al.* 2013). Nevertheless, the interpretation of the result should be made cautiously as the light exposure was not long enough (leaves remained in the chamber for 15 min) to ensure complete stomatal response. Gago *et al.* (2016) linked leaf gas exchange with leaf primary metabolism and reported that some sugars (mostly related to cell wall composition and structure; such as arabinose, xylose and galactose) had a significant effect on *g*_m_ but not *A* or *g*_sw_. However, cell wall properties are less likely to exert influence on *g*_m_ in short-term environmental changes.

The observation that *g*_m_ is lower under blue radiation than red radiation could be related to chloroplast movement away from blue radiation, the avoidance response, to avoid photodamage to the photosynthetic machinery (Kagawa and Wada 2002; Suetsugu and Wada 2007). The avoidance response would reduce *S*_c_ under high blue radiation, as reported by Tholen *et al.* (2008) in *Arabidopsis thaliana*. However, Loreto *et al.* (2009) showed that the rapid reduction of *g*_m_ under blue radiation in *Nicotiana* and *Platanus* leaves was faster than any possible chloroplast movements and the response was still observed after chloroplast movement inhibition. They suggested that the reduction of photosynthesis due to photochemical limitation under blue light might have, to some extent, affected *g*_m_. In our study, the radiation wavelength significantly affected the calculated *C*_c_, implying some extent of *g*_m_ limitation to photosynthesis under blue radiation. The response of *g*_m_ to blue radiation may have been caused by unknown factors affecting AQP-facilitated CO_2_ diffusion in the mesophyll (Kaldenhoff 2012).

Overall, these experiments demonstrate the considerable variability in measured *g*_m_ responses to both long-term and short-term changes in environmental conditions. Some of this variability is likely to result from measurement artefacts, because *g*_m_ is always the residual variation in measurements that include instrument noise. Part of the observed variability probably also results from the complex nature of the trait. That is, whether a response to a given environmental stimulus is present or not probably depends on the relative importance of the component resistance and if a given resistor is sensitive to a given stimulus.

## Conclusions

The present study showed that *g*_m_ varies between chickpea genotypes under ideal conditions and in response to growth conditions. This is the first study to examine the response of *g*_m_ to N_2_-fixing versus N-fed (uninoculated) legumes. Genotypes differed in the sensitivity of *g*_m_ to nitrogen source. Flip079C had higher *g*_m_ when fertilized with NH_4_NO_3_ than when nitrogen was fixed by *Rhizobium* inoculates. The *g*_m_ sensitivity to blue radiation was similar between the genotypes and growth environments. There was no clear indication of water availability effects on responses of *g*_m_ to PPFD. Genotypes differed in the effects of nitrogen source on the rapid response of *g*_m_ to PPFD. Little research has been done in the area of *g*_m_ regulation under different N sources, and future work should extend to examine a wide range of legumes and environments, and explore the underlying mechanisms of the results of this study in greater detail. The large *g*_m_ variability observed in our experiments indicates that it may be premature to recommend increased *g*_m_ as a target for improved productivity or water-use efficiency.

## Sources of Funding

This research was funded by the Australian Research Council and the Grains Research and Development Corporation through the ARC Industrial Transformation Research Centre, Legumes for Sustainable Agriculture. A.S. was supported by an Australian Postgraduate Award and International Postgraduate Research Support. T.N.B. acknowledges support from the Grains Research and Development Corporation and International Wheat Yield Partnership (US00082) and National Science Foundation (Award 1557906) and the USDA National Institute of Food and Agriculture, Hatch Project 1016439.

## Contributions by the Authors

A.S. and M.M.B conceived the study and designed the experiments. A.S. and E.L.L. carried out the experiments and analyzed the data. A.S. wrote the manuscript with input from all authors. M.M.B and T.N.B provided critical feedback and contributed to the interpretation of the results and to the final manuscript.

## Conflict of Interest

None declared.

## Supporting Information

The following additional information is available in the online version of this article—

Table S1. List of chickpea genotypes and their types used in Experiment 1.

Table S2. Effects of photosynthetic photon flux density (PPFD) on mesophyll conductance to CO_2_ (*g*_m_) across genotypes and treatments including radiation wavelength, water availability and nitrogen source in Experiments 2 and 3.

Table S3. Leaf gas exchange, online carbon isotope discrimination and mesophyll conductance values of 20 chickpea genotypes grown and measured under non-limiting controlled environmental conditions.

Table S4. Leaf gas exchange, online carbon isotope discrimination and mesophyll conductance values of the three chickpea genotypes grown under two nitrogen source treatments and measured under different photon flux densities.

Table S5. Leaf gas exchange, online carbon isotope discrimination and mesophyll conductance values of the three chickpea genotypes grown under well-watered or water-stressed conditions and measured under varying photon flux density and radiation wavelength.

Supplementary Table S1

Supplementary Table S2

Supplementary Table S3

Supplementary Table S4

Supplementary Table S5
