# Supplementary material for: The response of mesophyll conductance to short- and long-term environmental conditions in chickpea genotypes
Source: AoB Plants. 2018 Dec 11;11(1):ply073. doi: 10.1093/aobpla/ply073 (PMC6340285; doi:10.1093/aobpla/ply073)
Supplement: Supplementary Table S1 [file ply073_suppl_supplementary_table-s1.docx]

Table S1 List of chickpea genotypes and their types used in experiment 1

| **No.** | **Name** | **Type** |
| --- | --- | --- |
| 1 | Amethyst | Desi |
| 2 | BL1 | Desi |
| 3 | BL2 | Desi |
| 4 | BL3 | Desi |
| 5 | BL4 | Desi |
| 6 | BL5 | Desi |
| 7 | BL6 | Desi |
| 8 | BL7 | Desi |
| 9 | BL8 | Desi |
| 10 | BL9 | Desi |
| 11 | Jimbour | Desi |
| 12 | Kyabra | Desi |
| 13 | PBA Hattrick | Desi |
| 14 | PBA Slasher | Desi |
| 15 | Sonali | Desi |
| 16 | Tyson | Desi |
| 17 | Yorker | Desi |
| 18 | Flip 079C | Kabuli |
| 19 | Genesis 079C | Kabuli |
| 20 | PBA Monarch | Kabuli |
