# Supplementary material for: The response of mesophyll conductance to short- and long-term environmental conditions in chickpea genotypes
Source: AoB Plants. 2018 Dec 11;11(1):ply073. doi: 10.1093/aobpla/ply073 (PMC6340285; doi:10.1093/aobpla/ply073)
Supplement: Supplementary Table S2 [file ply073_suppl_supplementary_table-s2.docx]

Table S2 Effects of photosynthetic photon flux density (PPFD) on mesophyll conductance to CO_2_ (*g*_m_) across genotypes and treatments including radiation wavelength, water availability and nitrogen source in Experiment 2 and 3

| **Genotype/Treatment** | **Amethyst** | **PBA Slasher** | **Sonali** | **Flip 079C** |
| --- | --- | --- | --- | --- |
| **Red light-WW** | *g*_m_ = 0.00025*PPFD + 0.085 | *g*_m_ = 0.00022*PPFD + 0.106 | *g*_m_ = 0.00025*PPFD + 0.223 | - |
|  | R^2^=0.53, *p*<0.001 | R^2^=0.35, *p*=0.01 | R^2^=0.17, *p*=0.088 |  |
| **Red light-WS** | *g*_m_ = 0.00023*PPFD + 0.1 | *g*_m_ = 0.00031*PPFD + 0.0877 | *g*_m_ = 0.00020*PPFD + 0.0769 | - |
|  | R^2^=0.29, *p*=0.026 | R^2^=0.51, *p*=0.003 | R^2^=0.33, *p*=0.026 |  |
| **Blue light-WW** | *g*_m_ = 0.00015*PPFD + 0.044 | *g*_m_ = 0.00015*PPFD + 0.0235 | *g*_m_ = 0.00031*PPFD - 0.0158 | - |
|  | R^2^=0.52, *p*=0.003 | R^2^=0.60, *p*<0.001 | R^2^=0.49, *p*=0.002 |  |
| **Blue light-WS** | *g*_m_ = 0.00015*PPFD + 0.054 | *g*_m_ = 0.00016*PPFD + 0.0395 | *g*_m_ = 0.00011*PPFD + 0.0569 | - |
|  | R^2^=0.32, , *p*=0.015 | R^2^=0.19, *p*=0.124 | R^2^=0.18, *p*=0.154 |  |
| **N-fed** | - | *g*_m_ = 0.00019*PPFD + 0.129 | *g*_m_ = 0.00043*PPFD + 0.135 | *g*_m_ = 0.00029*PPFD + 0.139 |
|  |  | R^2^=0.48, *p*=0.004 | R^2^=0.79, *p*<0.001 | R^2^=0.40, *p*<0.001 |
| **N-fixed** | - | *g*_m_ = 0.00024*PPFD + 0.075 | *g*_m_ = 0.00023*PPFD + 0.241 | *g*_m_ = 0.00015*PPFD + 0.0994 |
|  |  | R^2^=0.42, *p*<0.001 | R^2^=0.16, *p*=0.077 | R^2^=0.17, *p*=0.038 |

Well-watered conditions (WW), Water-stressed conditions (WS), Inoculated with *Rhizobium inocula* without mineral N supply (N-fixed) and Uninoculated, supplied with 2.5 mM NH_4_NO_3_ (N-fed). The regression equations between *g*_m_ and PPFD, R^2^ and *p* value are given.
